# Campaigning for Organ Donation at Mosques

**DOI:** 10.1007/s10730-016-9302-3

**Published:** 2016-03-03

**Authors:** Mohamed Y. Rady, Joseph L. Verheijde

**Affiliations:** 1 Department of Critical Care Medicine, Mayo Clinic Hospital, 5777 East Mayo Blvd, Phoenix, AZ 85054 USA; 2 Department of Physical Medicine and Rehabilitation, Mayo Clinic, 13400 E Shea Blvd, Scottsdale, AZ 85259 USA

**Keywords:** Death, Do-no-harm principle, End-of-life care, Living donors, Organ donation, Religion

## Abstract

There is a trend of recruiting faith leaders at mosques to overcome religious barriers to organ donation, and to increase donor registration among Muslims. Commentators have suggested that Muslims are not given enough information about organ donation in religious sermons or lectures delivered at mosques. Corrective actions have been recommended, such as funding campaigns to promote organ donation, and increasing the availability of organ donation information at mosques. These actions are recommended despite published literature expressing safety concerns (i.e., do no harm) in living and end-of-life organ donation. Living donors require life-long medical follow-up and treatment for complications that can appear years later. Scientific and medical controversies persist regarding the international guidelines for death determination in end-of-life donation. The medical criteria of death lack validation and can harm donors if surgical procurement is performed without general anesthesia and before biological death. In the moral code of Islam, the prevention of harm holds precedence over beneficence. Moral precepts described in the Quran encourage Muslims to be beneficent, but also to seek knowledge prior to making practical decisions. However, the Quran also contains passages that demand honesty and truthfulness when providing information to those who are seeking knowledge. Currently, information is limited to that which encourages donor registration. Campaigning for organ donation to congregations in mosques should adhere to the moral code of complete, rather than selective, disclosure of information. We recommend as a minimal standard the disclosure of risks, uncertainties, and controversies associated with the organ donation process.

## Introduction

Persistent medical, ethical, and legal challenges have hindered the global acceptance of organ donation and transplantation (Nature 2009; Rady et al. 2010). Cultural and religious barriers have also been identified as factors influencing societal attitudes toward organ donation (Tumin et al. 2013, 2014). Within religious communities, it is widely held to be true that religious authorities convey positions and directives on complex societal issues that are congruent with the theology and moral values of those communities. Knowledge and comprehension of empirical facts, as currently known, constitute additional elements in the decision-making process about the moral permissibility of an action or a class of actions.

Elsewhere, concerns have been expressed about a growing trend of recruiting faith leaders to promote organ donation in Muslim communities (Rady and Verheijde 2014). Members of religious communities tend to accept that faith leaders are honest and transparent, and that their authoritative positions and opinions are reflective of the moral values within their respective belief systems. However, sometimes that trust is put to the test. The moral legitimacy of organ donation represents one of those times. Faith leaders are not necessarily up-to-date on the empirical medical and scientific facts relevant to living organ donation. Nor are they well informed on the controversies surrounding the definition of death in end-of-life organ donation. Both of these knowledge insufficiencies are likely to negatively impact the moral legitimacy of organ donation, or at least to call its legitimacy into question. Nevertheless, the information shared by faith leaders during sermons can have a considerable impact on practical and moral decision making of members of the religious community. Marketing efforts directed toward collaboration with faith leaders in the pursuit of promoting organ donation should be respectful of the special status that faith leaders have on the moral decision-making of observant Muslims.

Tumin and colleagues (2015b) surveyed 653 Malaysian Muslims from 82 mosques in Kuala Lumpur regarding their knowledge of and access to information about organ donation. They reported that 54–64 % of respondents did not have access to information at either religious sermons or lectures at mosques. In an effort to correct this circumstance, Tumin et al. (2015b) have recommended that governmental funding be made available, and that faith leaders be encouraged to participate in campaigns aimed at increasing “organ donation activities at mosques” and “providing accurate information to the Malaysian Muslim community.” However, “accurate information” about living and end-of-life organ donation is then limited to include only the information that encourages donor registration among citizens (Tumin et al. 2015a). Within such a context, the recommendation of recruiting faith leaders and promoting organ donation at mosques as a public policy buttresses the problem of trustworthiness of information. Information distributed by faith leaders during sermons generates different levels of expectations regarding trustworthiness and truthfulness compared to ordinary marketing campaigns. The call for allocation of governmental funds to implement this public policy also implies the unconditional endorsement of living and end-of-life organ donation by governmental officials, policy makers, and religious authorities. This has serious negative consequences in Muslim communities. In this article, we comment on the medical, legal, moral, and religious harm of limited disclosure of information to potential donors. The moral code of Islam places a high priority on the do-no-harm principle in terms of a person’s religion, life, mind, property, and progeny (Rady and Verheijde 2014). Islam encourages individuals to be beneficent, but also to seek knowledge prior to making these practical decisions. We argue that selective disclosure of promotional information about organ donation in mosques directly conflicts with at least two principles in the moral code: (1) truthfulness and honesty in information disclosure and (2) precedence of prevention of harm over beneficence. We conclude with a recommendation on the minimal standard of prerequisite information disclosure, so as to allow a reasonable person to make rational decisions. We urge that this standard is maintained in all educational campaigns. Failure to do so violates the trust of Muslims in faith leaders, and degrades mosques from places of worship into advertising venues for organ donation.

## Living Organ Donation

There is a growing body of medical literature expressing serious concerns about the do-no-harm principle and safety in living organ donation. Incomplete long-term follow-up and data collection about living donors have limited the accuracy of previously reported outcomes following kidney donation (Schold et al. 2015; Ross 2015; Lentine et al. 2015). Kidney donors have an increased lifetime risk of developing kidney disease and cardiovascular complications (Mjoen et al. 2014; Yilmaz et al. 2015). Mjoen and colleagues (2014) have reported an increased risk of all-cause mortality, cardiovascular mortality, and end-stage kidney disease in donors compared to control subjects over a median follow-up of 15 years. The lifetime risk of end-stage kidney disease is increased in kidney donors compared to health-matched non-donors within a median follow-up of 7.6 years (Muzaale et al. 2014). Certain demographic and health characteristics have been shown to increase the risk of end-stage kidney disease by 3.5–5.3 times in donors compared to matched non-donors (Grams et al. 2016). Gestational hypertension and preeclampsia have been reported in 11 % of pregnancies in 85 women kidney donors compared to 5 % in pregnancies in health-matched non-donor women (Garg et al. 2015). The sample size in this study was too small to detect an associated increase in maternal and fetal morbidity, as well as mortality. Living donation has also been associated with adverse psychosocial outcomes because of changes in self-perception of body image (DuBay et al. 2010). An increased incidence of depression in donors has been attributed to pre-donation psychosocial disorders, ethnicity, young age, moral obligation to donation, prolonged recovery time, and burdensome financial stressors (Jowsey et al. 2014). Financial stressors include expenses for prolonged treatment and follow-up due to unexpected post-donation medical complications and delayed return to daily activities and employment (Klarenbach et al. 2014). A delay in or inability to return to pre-donation employment because of persistent physical, mental, and/or emotional impairment can also contribute to economic hardship (Rodrigue et al. 2016). The persistence of psychosocial, psychological, and financial difficulties has been reported in donors at an average of 17 years post-donation (Jacobs et al. 2015). Preemptive strategies have been recommended in all living donors to mitigate these long-term adverse psychosocial and financial consequences (Dew et al. 2014).

Living organ donation increases the utilization of medical care in resource-limited healthcare systems. This requires advanced allocation of health care resources at the national level to avoid unsafe practices in living donation (Van Assche et al. 2015). Kidney donors require life-long screening and treatment of hypertension, kidney disease, and cardiovascular complications that can develop years after donation (Newell et al. 2016). The lack of necessary health care resources and appropriate post-donation medical care will negatively affect the quality of life and lifespan of living donors. Transplant recipients generally also require intensive utilization of health care resources because of life-long medical follow-up and treatment of complications that can develop from immunological rejection and adverse effects of immunosuppression medication. The 2014 US total estimated cost of medical care for 27,790 transplant recipients of single- or multiple-solid organs was in excess of $ 15 billion (Hanson and Bentley 2014). This cost estimate included the transplant and the first six months of subsequent medical care. The transplanted organs were procured from deceased donors and, thus, the cost of medical care of living donors over an entire lifespan remains unknown. Therefore, providing optimal quality of medical care to living donors and recipients will likely strain national healthcare resources in some countries.

## End-of-life Organ Donation

Scientific concerns have also been expressed about harm to donors in end-of-life organ donation (Joffe 2009; Joffe et al. 2011; Halpern 2014; Nair-Collins 2015). International guidelines for death determination have been developed, in collaboration with the World Health Organization, to increase the global supply of transplantable organs at the end of life (Shemie et al. 2014). These guidelines redefine death as the:

*permanent* cessation of brain function (loss of capacity for consciousness and brainstem reflexes), which occurs along two pathways: (1) permanent absence of circulation or (2) subsequent to a catastrophic brain injury, each discerned through a specific set of medical criteria and clinical and laboratory tests—two entrances, one end point (Shemie et al. 2014) [emphasis added].

There are biological and practical differences between “*permanent* cessation (*will not* return) and *irreversible* cessation (*cannot* return)” of vital functions (Bernat et al. 2010) [emphasis added]. The loss of consciousness, brainstem functions, or circulation is considered permanent when these vital functions *will not* resume spontaneously, though they can resume through medical intervention. In contrast, ceased vital functions are considered irreversible if they *cannot* resume despite medical intervention. From a biological perspective, permanent cessation of vital functions signifies the beginning of the dying process, whereas irreversible cessation of vital functions signifies the completion of the dying process. From a practical perspective, the cessation of circulation or brain function is potentially reversible by intervention during organ procurement. The international guidelines on death determination have constructed medical criteria that do not fulfill the necessary condition of irreversibility of ceased vital functions. These criteria have not been scientifically validated to ensure the biological uniformity of death determination. It is not surprising that major disagreement exists within the international medical community on the validity and acceptability of the newly defined criteria of death (Kuiper and Kompanje 2014; Rusinova and Simek 2014; Wahlster et al. 2015; Bernat 2015). Although these death criteria enable the procurement of transplantable organs, they can also harm donors at the end of life (Iltis 2015; Nair-Collins 2015). The foremost harm is that organs are surgically procured from donors without the necessary general anesthesia prior to biological death. Brain functions that have not ceased irreversibly can resume during surgical procurement. Resumption of movements has been reported in about 50 % of donors (Saposnik et al. 2009). These movements have been generally dismissed as spinal cord reflexes despite the fact that they can also be mediated at the brainstem. Indeed, movements of donors are commonly observed in response to nociceptive stimuli during surgical procurement and are routinely masked by administering neuromuscular-blocking drugs to induce paralysis (Anderson et al. 2015). However, analgesic and hypnotic drugs are not administered concurrently to relieve pain and suppress residual awareness that may be present in these donors.

The construct of death criteria that fails to assure the biological irreversibility and, thus, the uniformity of death determination has legal consequences. Such a construct can result in an erroneous determination of death when certain brain functions are still present or recoverable. The US case of Jahi McMath illustrates the failure of current death criteria to ensure biological uniformity in death determination (Crippen 2014). In such cases, legal proceedings have challenged the ambiguity in the process of medically determining when a human being is dead (Luce 2015). The opinion of the Supreme Court of Nevada (2015) in *Hailu vs Prime Healthcare* has emphasized that the medical criteria of death must comply with the legal standard of the Uniform Determination of Death Act (UDDA):

[f]or legal and medical purposes, a person is dead if the person has sustained *an irreversible cessation of:* (a) Circulatory and respiratory functions; or (b) *All functions* of the person’s *entire brain, including his or her brain stem*…(emphasis added)*….* the UDDA sought to achieve greater uniformity in making such important and profound medical determinations [emphasis in the original document].

The Supreme Court of Nevada (2015) has re-affirmed that “[t]hough courts defer to the medical community to determine the applicable criteria to measure brain functioning, it is the duty of the law to establish the applicable standard that said criteria must meet.” Therefore, the Supreme Court of Nevada has indirectly rejected the death criteria in the international guidelines developed by Shemie et al. (2014). First, the legal standard demands not only “permanent” but “irreversible” cessation of functions so that the ceased vital functions are not reversible by subsequent medical intervention. Second, the legal standard requires that cessation of functions must include the “entire brain, including his or her brainstem” rather than only a subset of brain functions, i.e., the “capacity for consciousness and brainstem reflexes” or, alternatively, the cessation of both “circulatory and respiratory (brainstem) functions” rather than “circulation” only. The discord between the medical and legal standards will result in an incorrect death determination, which would also mean that the surgical procurement is the proximate cause of death in those who are donating organs at the end of life. The legal code prohibits homicide by organ procurement (Verheijde et al. 2009; Harrington 2009).

There are serious consequences of an erroneous determination of death in Islam (Rady and Verheijde 2013). Religions across the world, including Islam, only permit organ donation conditionally on the premises that organ procurement: (1) is not itself the cause of death, and (2) complies with their established moral and religious values (The Lancet 2011; Choong 2013). Scientific evidence has not validated the current criteria of death. Therefore, the absence of validation casts a level of reasonable doubt on proponents’ claims that procurement is not the proximate cause of the donor’s death. Homicide by organ procurement has severe repercussions in Islam:

And do not kill yourselves (nor kill one another). Surely, Allah [God] is Most Merciful to you. (29) And whoever commits that through aggression and injustice, We shall cast him into the Fire, and that is easy for Allah. (30) (The Quran Chapter 4: verses 29-30)

Furthermore, various pre-procurement interventions may pose challenges to the moral and religious values of at least some donors (Rady et al. 2009). The goals of these interventions are to orchestrate a planned time, place, and method of death in order to secure the procurement of transplantable organs. Life-support interventions are initiated and maintained for the sole purpose of organ preservation until surgical procurement is accomplished (Najafizadeh et al. 2012). Interventions that can artificially manipulate the dying process and/or violate the physical integrity of the body are generally harmful at the end of life and are prohibited in Islam (Rady and Verheijde 2013).

## Organ Donation Marketing Campaigns

Marketing and educational campaigns are specifically designed to improve donor recruitment (Rady et al. 2012, 2013). These campaigns are directed at the general public and generally communicate incomplete and selective information about the actual process and consequences of organ donation (Milligan et al. 2012). They emphasize the benefit of organ transplantation to recipients, but are universally silent about the potential harm to donors (Milligan et al. 2012; Rady et al. 2012; Iltis 2015).

Consent to end-of-life donation is obtained through either enrollment in a national donor registry, or a surrogate agreement at the time when organ procurement is de facto being proposed. The medical procedures that are performed at the end of life on donors to preserve organs for transplantation, the criteria used to determine death, and the surgical procedures performed to procure organs are not disclosed. Without full disclosure of relevant information, individuals are not allowed the opportunity to make fully-informed decisions. Relevant information pertains, at a minimum, to disclosure of persistent medical controversies about the definition of death and the criteria by which death is determined (Iltis 2015). Other information commonly not disclosed is related to changes in the procurement policies and processes, including the seemingly innocuous modification from “consent to donation” to “authorization of donation” (Rady et al. 2012, 2013). Authorization arguably resolves the issue of providing sufficient information of relevant material to the decision-making agent because authorizing simply refers to the process of giving someone permission, in this case to procure organs *after death* (Iltis 2015). However, few, if any, of the potential donors are made aware of the wording change when registering as an organ donor.

There are also serious deficiencies in the living donation consenting process. The deficiencies are related to major gaps in the content, accuracy, and communication of essential information to potential donors (Biancone et al. 2016; Cozzi et al. 2016). The failure to ascertain donor comprehension on the information about future risks and to recognize the consequences of donation on their daily lives negates the claim that consent is fully informed and truly free of coercion. Some living donors have felt pressured to consent to donate because of moral obligation (Gordon et al. 2011; Valapour et al. 2011; Kortram et al. 2014). In a survey of 262 living kidney donors, “only 69 % understood the psychological risks of donation; 52 % the long-term medical risks of donation; and 32 % the financial risks of donation” (Valapour et al. 2011). In the same survey, 40 % of living donors reported “feeling some pressure to donate.” Donors who have consented to living liver donation also reported that they were inadequately informed about future risks and unexpected complications (Gordon et al. 2011). The inconsistency among US and European transplant centers in disclosing information about living donation risks to donors has called into question the validity of informed consent (Kortram et al. 2014). Critical information that is generally overlooked pertains to the long-term physical, psychosocial, and financial consequences on living donors. Providing incomplete information violates regulatory requirements and compromises moral obligations—the foundational elements of ensuring true informed consent. This ultimately weakens the trust of the global community in the organ donation and transplantation practice.

## Compliance with the Moral Code of Islam

The moral code of Islam is set forth in the Quran (revelation from God to man) and the Sunnah (the tradition of the Prophet Muhammad: what he said, what he did, and what he saw and approved of during his lifetime) and is intended to apply regardless of time and place (Rady and Verheijde 2014). According to the Quran, God is the Creator of man, and He alone grants life and takes life at a pre-ordinated time and place: “No person knows what he will earn tomorrow, and no person knows in what land he will die. Verily, Allah[God] is All-Knower, All-Aware” (The Quran Chapter 31: verse 34). God has exalted the creation of man and dignified him above all other creations:

And indeed We have honoured the Children of Adam, and We have carried them on land and sea, and have provided them with lawful good things, and have preferred them above many of those whom We have created with a marked preferment (The Quran Chapter 17: verse 70).

The moral code is centered on upholding the value and dignity not only of human life, but also of the physical body created by God. The prevention of harm is prioritized over beneficence; the do-no-harm principle is prescribed in the Quran and the Sunnah: “and do not throw yourselves into destruction and do good” (The Quran Chapter 2: verse 195). And the Prophet said: “[t]here should be neither harming (*darar*) nor reciprocating harm (*dirar*)” (The Hadith of the Prophet Muhammad, Hadith 32 of 40 Hadiths Nawawi). Each individual is held accountable in the Hereafter for each and every act in his (her) own worldly life. If living donors can potentially suffer long-term physical, mental, emotional and/or financial harm, the moral code would not sanction such a potentially self-harming act. A dying person is at their most vulnerable time since birth. The moral code expects that all acts in end-of-life care are conforming to and upholding the respect for the sanctity of both human life and the physical body. Invasive perimortem procedures are necessary for preserving and procuring transplantable organs in end-of-life donation (Rady et al. 2009). However, these donation-related procedures are not beneficial, but rather are harmful and interfere with important religious rituals at the end of life (Rady and Verheijde 2013).

Mosques are primarily places of worship. The Quran states: “And the mosques are for Allah [God] (alone): so invoke not anyone along with Allah” (The Quran Chapter 72: verse 18). Educational sermons and campaigns are permitted at mosques, but they must adhere to the Quranic command to be truthful and honest. Utilizing mosques as places for campaigning and educating about organ donation is permissible only if accurate and complete information is disclosed to worshippers so as to ensure that the decisions they make are informed and will be made voluntarily. We re-emphasize here the fact that the Islamic moral code considers it a moral obligation that both the harm and the benefit of organ donation be fully disclosed to potential donors. The Quran warns those who are utilizing mosques as places for misinforming Muslims, promoting harmful practices, and corrupting places of worship:

And as for those who put up a mosque by way of harm and disbelief, and to disunite the believers, and as an outpost for those who warred against Allah [God] and His Messenger (Muhammad SAW) aforetime, they will indeed swear that their intention is nothing but good. Allah bears witness that they are certainly liars (The Quran Chapter 9: verse 107);

And who are more unjust than those who forbid that Allah’s [God] Name be glorified and mentioned much (i.e. prayers and invocations, etc.) in Allah’s mosques and strive for their ruin? It was not fitting that such should themselves enter them (Allah’s Mosques) except in fear. For them there is disgrace in this world, and they will have a great torment in the Hereafter (The Quran Chapter 2: Vesre 114).

For the recommendation of campaigning for donor recruitment at mosques to be acceptable, it must adhere to the moral values of truthfulness and honesty when providing information to others. The minimal criteria for disclosure must include information that a reasonable individual should expect and is able to understand before making a decision on organ donation in general. Such a decision must be consistent with avoiding personal harm and upholding individual values and preferences. The individual must also fully comprehend and accept the negative consequences and harm of that decision. We recommend a minimal standard of prerequisite information that should be disclosed to potential living and end-of-life donors in campaigns at mosques and in Muslim communities (Table 1). Limiting the disclosure only to favorable information about donation will violate the trust of worshippers at mosques.

**Table 1:** The minimal standard of prerequisite information that should be disclosed in organ donation campaigns at mosques and in Muslim communities

Prerequisite information in living donation	Prerequisite information in end-of-life donation
Comprehensive pre-donation physical, psychological, social and financial evaluation before approval	Address conflicts between end-of-life care and perimortem organ preservation
Enrollment in a national living-donor registry for life-long medical follow-up	Disclose the scientific, medical, and religious controversies about the determination of death
Reliable data collection about preexisting health risk factors and subsequent complications in donors	Explain that transplantable organs will be procured before biological death
Life-long screening for development of delayed post-donation physical and psychosocial complications	Describe that organ procurement is performed without general anesthesia
Availability and affordability of healthcare resources for treatment of post-donation short and long-term physical consequences	Expand on the conflict between donation-related procedures and religious rituals at the end of life
Provision of post-donation psychological and psychiatric counselling	
Practical strategies for mitigating post-donation financial consequences and economic hardship	
Independent tracking and comprehensive reporting of outcome measures in all donors	

## Conclusions

Contemporary scientific literature has raised several safety concerns for both living and end-of-life organ donation. The moral code of Islam prioritizes the prevention of harm over beneficence. The do-no-harm principle in the moral code applies to a person’s religion, life, mind, property, and progeny. Islam encourages individuals to be beneficent, but also to seek knowledge prior to making these practical decisions. Those providing information about organ donation to potential donors are obligated to do so with full disclosure of harm and benefit. Therefore, for campaigns aimed at congregations in mosques to be acceptable, they must minimally adhere to the command of truthfulness and honesty in full disclosure of information about organ donation. Failure to do so violates the trust of Muslims in their faith leaders and degrades mosques from places of worship into propaganda venues for organ donation.

## References

[CR2] Anderson TA, Bekker P, Vagefi P (2015). Anesthetic considerations in organ procurement surgery: A narrative review. Canadian Journal of Anesthesia/Journal canadien d’anesthésie.

[CR3] Bernat JL (2015). Comment: Is international consensus on brain death achievable?. Neurology.

[CR4] Bernat JL, Capron AM, Bleck TP, Blosser S, Bratton SL, Childress JF, DeVita MA, Fulda GJ, Gries CJ, Mathur M, Nakagawa TA, Rushton CH, Shemie SD, White DB (2010). The circulatory-respiratory determination of death in organ donation. Critical Care Medicine.

[CR5] Biancone L, Cozzi E, López-Fraga M, Nanni-Costa A (2016). Long-term outcome of living kidney donation: Position paper of the European Committee on Organ Transplantation (CD-P-TO), Council of Europe. Transplant International.

[CR6] Choong KA (2013). Organ procurement: A case for pluralism on the definition of death. The Journal of Medical Law and Ethics.

[CR7] Cozzi E, Biancone L, López-Fraga M, Nanni-Costa A (2016). Long-term outcome of living kidney donation: Position Paper of the European Committee on Organ Transplantation, Council of Europe. Transplantation.

[CR8] Crippen D (2014). Changing interpretations of death by neurologic criteria: The McMath case. Journal of Critical Care.

[CR9] Dew MA, Myaskovsky L, Steel JL, Dimartini AF (2014). Managing the psychosocial and financial consequences of living donation. Current Transplantation Reports.

[CR10] DuBay DA, Holtzman S, Adcock L, Abbey SE, Greenwood S, Macleod C, Kashfi A, Renner EL, Grant DR, Levy GA, Therapondos G (2010). Cosmesis and body image after adult right lobe living liver donation. Transplantation.

[CR11] Garg AX, Nevis IF, McArthur E, Sontrop JM, Koval JJ, Lam NN, Hildebrand AM, Reese PP, Storsley L, Gill JS, Segev DL, Habbous S, Bugeja A, Knoll GA, Dipchand C, Monroy-Cuadros M, Lentine KL (2015). Gestational hypertension and preeclampsia in living kidney donors. New England Journal of Medicine.

[CR12] Gordon EJ, Daud A, Caicedo JC, Cameron KA, Jay C, Fryer J, Beauvais N, Skaro A, Baker T (2011). Informed consent and decision-making about adult-to-adult living donor liver transplantation: A systematic review of empirical research. Transplantation.

[CR13] Grams ME, Sang Y, Levey AS, Matsushita K, Ballew S, Chang AR, Chow EKH, Kasiske BL, Kovesdy CP, Nadkarni GN, Shalev V, Segev DL, Coresh J, Lentine KL, Garg AX (2016). Kidney-failure risk projection for the living kidney-donor candidate. New England Journal of Medicine.

[CR14] Halpern SD (2014). Donation after circulatory determination of death: Time for transparency. Annals of Emergency Medicine.

[CR15] Hanson, S. G., & Bentley, T. S. (2014). Milliman Research Report of 2014 U.S. organ and tissue transplant cost estimates and discussion. http://www.milliman.com/insight/research/health/2014-U_S_-organ-and-tissue-transplant-cost-estimates-and-discussion/ Accessed 24 February 2016.

[CR16] Harrington MM (2009). The thin flat line: Redefining who is legally dead in organ donation after cardiac death. Issues in Law and Medicine.

[CR17] Iltis AS (2015). Organ donation, brain death and the family: Valid informed consent. The Journal of Law, Medicine & Ethics.

[CR18] Jacobs CL, Gross CR, Messersmith EE, Hong BA, Gillespie BW, Hill-Callahan P, Taler SJ, Jowsey SG, Beebe TJ, Matas AJ, Odim J, Ibrahim HN (2015). Emotional and financial experiences of kidney donors over the past 50 Years: The RELIVE study. Clinical Journal of the American Society of Nephrology.

[CR19] Joffe AR (2009). Brain death is not death: A critique of the concept, criterion, and tests of brain death. Reviews in the Neurosciences.

[CR20] Joffe AR, Carcillo J, Anton N, deCaen A, Han Y, Bell M, Maffei F, Sullivan J, Thomas J, Garcia-Guerra G (2011). Donation after cardiocirculatory death: A call for a moratorium pending full public disclosure and fully informed consent. Philosophy, Ethics, and Humanities in Medicine.

[CR21] Jowsey SG, Jacobs C, Gross CR, Hong BA, Messersmith EE, Gillespie BW, Beebe TJ, Kew C, Matas A, Yusen RD, Hill-Callahan M, Odim J, Taler SJ, The R.S.G. (2014). Emotional well-being of living kidney donors: Findings from the RELIVE Study. American Journal of Transplantation.

[CR22] Klarenbach S, Gill JS, Knoll G, Caulfield T, Boudville N, Prasad GVR, Karpinski M, Storsley L, Treleaven D, Arnold J, Cuerden M, Jacobs P, Garg AX, for the Donor Nephrectomy Outcomes Research Network (2014). Economic consequences incurred by living kidney donors: A Canadian multi-center prospective study. American Journal of Transplantation.

[CR23] Kortram K, Lafranca JA, IJzermans JNM, Dor FJMF (2014). The need for a standardized informed consent procedure in live donor nephrectomy: A systematic review. Transplantation.

[CR24] Kuiper M, Kompanje EO (2014). Only a very bold man would attempt to define death. Intensive Care Medicine.

[CR25] Lancet The (2011). Religion, organ transplantation, and the definition of death. The Lancet.

[CR26] Lentine KL, Schnitzler MA, Garg AX, Xiao H, Axelrod D, Tuttle-Newhall JE, Brennan DC, Segev DL (2015). Race, relationship and renal diagnoses after living kidney donation. Transplantation.

[CR27] Luce JM (2015). The uncommon case of Jahi McMath. Chest.

[CR28] Milligan E, Winch S, Adams R (2012). Marketing to register organ donors may circumvent principles of informed consent. British Medical Journal.

[CR29] Mjoen G, Hallan S, Hartmann A, Foss A, Midtvedt K, Oyen O, Reisater A, Pfeffer P, Jenssen T, Leivestad T, Line P-D, Ovrehus M, Dale DO, Pihlstrom H, Holme I, Dekker FW, Holdaas H (2014). Long-term risks for kidney donors. Kidney International.

[CR30] Muzaale AD, Massie AB, Wang M-C, Montgomery RA, McBride MA, Wainright JL, Segev DL (2014). Risk of end-stage renal disease following live kidney donation. JAMA, the Journal of the American Medical Association.

[CR31] Nair-Collins M (2015). Taking science seriously in the debate on death and organ transplantation. Hastings Center Report.

[CR32] Najafizadeh K, Ghobadi O, Ghorbani F, Shafaghi S, Radpei B, Shojaei SP (2012). Transfer protocol of brain-dead patients to specialized donor management unit. Transplantation.

[CR33] Nature (2009). Delimiting death. Nature.

[CR34] Newell KA, Formica RN, Gill JS (2016). Engaging living kidney donors in a new paradigm of postdonation care. American Journal of Transplantation.

[CR35] Rady MY, McGregor JL, Verheijde JL (2012). Mass media campaigns and organ donation: Managing conflicting messages and interests. Medicine, Health Care and Philosophy.

[CR36] Rady MY, McGregor JL, Verheijde JL (2013). Transparency and accountability in mass media campaigns about organ donation: A response to Morgan and Feeley. Medicine, Health Care and Philosophy.

[CR37] Rady MY, Verheijde JL (2013). Brain-dead patients are not cadavers: The need to revise the definition of death in Muslim communities. HEC Fourm: HealthCare Ethics Committee Forum.

[CR38] Rady MY, Verheijde JL (2014). The moral code in Islam and organ donation in Western countries: Reinterpreting religious scriptures to meet utilitarian medical objectives. Philosophy, Ethics, and Humanities in Medicine.

[CR39] Rady MY, Verheijde JL, Ali MS (2009). Islam and end-of-life practices in organ donation for transplantation: New questions and serious sociocultural consequences. HEC Fourm: HealthCare Ethics Committee Forum.

[CR40] Rady MY, Verheijde JL, McGregor JL (2010). Scientific, legal, and ethical challenges of end-of-life organ procurement in emergency medicine. Resuscitation.

[CR100] Rodrigue JR, Schold JD, Morrissey P, Whiting J, Vella J, Kayler LK (2016). Direct and indirect costs following living kidney donation: Findings from the KDOC study. American Journal of Transplantation.

[CR41] Ross LF (2015). Living kidney donors and ESRD. American Journal of Kidney Diseases.

[CR42] Rusinova K, Simek J (2014). Should we relax the definition of death or the dead donor rule?. Intensive Care Medicine.

[CR43] Saposnik G, Basile VS, Young GB (2009). Movements in brain death: A systematic review. Canadian Journal of Neurological Sciences.

[CR44] Schold JD, Buccini LD, Rodrigue JR, Mandelbrot D, Goldfarb DA, Flechner SM, Kayler LK, Poggio ED (2015). Critical factors associated with missing follow-up data for living kidney donors in the United States. American Journal of Transplantation.

[CR45] Shemie S, Hornby L, Baker A, Teitelbaum J, Torrance S, Young K, Capron A, Bernat J, Noel L (2014). International guideline development for the determination of death. Intensive Care Medicine.

[CR46] The Hadith of the Prophet Muhammad. http://sunnah.com/. Accessed 24 February 2016.

[CR1] The Quran (Mohsin Khan-English Translation). http://www.quranexplorer.com/Quran/. Accessed 24 February 2016.

[CR47] The Supreme Court of Nevada (2015). Hailu vs Prime Healthcare Case No. 68531. http://caseinfo.nvsupremecourt.us/document/view.do?csNameID=36740&csIID=36740&deLinkID=530188&sireDocumentNumber=15-34970. Accessed 24 February 2016.

[CR48] Tumin M, Mohd Satar N, Zakaria RH, Raja Ariffin RN, Soo-Kun L, Kok-Peng N (2015). Determinants of willingness to become organ donors among dialysis patients’ family members. Urology Journal.

[CR49] Tumin M, Noh A, Mohd Satar N, Chin-Sieng C, Soo-Kun L, Abdullah N, Kok-Peng N (2013). Organ donation in Muslim countries: The case of Malaysia. Annals of Transplantation.

[CR50] Tumin M, Raja Ariffin RN, Mohd Satar N, Abdullah N, Wan Md Adnan WAH, Ismail AZ, Che Soh M (2015). Organ donation among Malaysian Muslims: The role of mosques. Annals of Transplantation.

[CR51] Tumin M, Rasiah R, Noh A, Satar NM, Chong C-S, Lim S-K, Ng K-P (2014). Living kidney donation: The importance of public education. Clinical Transplantation.

[CR52] Valapour M, Kahn JP, Bailey RF, Matas AJ (2011). Assessing elements of informed consent among living donors. Clinical Transplantation.

[CR53] Van Assche K, Sterckx S, Lennerling A, Mamode N, Citterio F, Frunza M, Zuidema WC, Burnapp L, Weimar W, Dor FJMF (2015). The relevance of directive 2010/53/EU for living organ donation practice: An ELPAT view. Transplantation.

[CR54] Verheijde JL, Rady MY, McGregor JL (2009). Brain death, states of impaired consciousness, and physician-assisted death for end-of-life organ donation and transplantation. Medicine, Health Care and Philosophy.

[CR55] Wahlster S, Wijdicks EFM, Patel PV, Greer DM, Hemphill JC, Carone M (2015). Brain death declaration: Practices and perceptions worldwide. Neurology.

[CR56] Yilmaz BA, Caliskan Y, Yilmaz A, Ozkok A, Bilge AK, Deniz G, Sariyar M, Yildiz A (2015). Cardiovascular-renal changes after kidney donation: One-year follow-up study. Transplantation.

